# Yeast Models for Amyloids and Prions: Environmental Modulation and Drug Discovery

**DOI:** 10.3390/molecules24183388

**Published:** 2019-09-18

**Authors:** Tatiana A. Chernova, Yury O. Chernoff, Keith D. Wilkinson

**Affiliations:** 1 Department of Biochemistry, Emory University School of Medicine, Atlanta, GA 30322, USA; 2 School of Biological Sciences, Georgia Institute of Technology, Atlanta, GA 30332, USA; yury.chernoff@biology.gatech.edu; 3 Laboratory of Amyloid Biology, St. Petersburg State University, St. Petersburg 199034, Russia

**Keywords:** amyloid, prion, chaperone, ubiquitin, heat shock, environmental factors, neurodegenerative disease, drug discovery

## Abstract

Amyloids are self-perpetuating protein aggregates causing neurodegenerative diseases in mammals. Prions are transmissible protein isoforms (usually of amyloid nature). Prion features were recently reported for various proteins involved in amyloid and neural inclusion disorders. Heritable yeast prions share molecular properties (and in the case of polyglutamines, amino acid composition) with human disease-related amyloids. Fundamental protein quality control pathways, including chaperones, the ubiquitin proteasome system and autophagy are highly conserved between yeast and human cells. Crucial cellular proteins and conditions influencing amyloids and prions were uncovered in the yeast model. The treatments available for neurodegenerative amyloid-associated diseases are few and their efficiency is limited. Yeast models of amyloid-related neurodegenerative diseases have become powerful tools for high-throughput screening for chemical compounds and FDA-approved drugs that reduce aggregation and toxicity of amyloids. Although some environmental agents have been linked to certain amyloid diseases, the molecular basis of their action remains unclear. Environmental stresses trigger amyloid formation and loss, acting either via influencing intracellular concentrations of the amyloidogenic proteins or via heterologous inducers of prions. Studies of environmental and physiological regulation of yeast prions open new possibilities for pharmacological intervention and/or prophylactic procedures aiming on common cellular systems rather than the properties of specific amyloids.

## 1. Protein Misfolding Diseases

Amyloids are highly ordered fibrous protein aggregates in a cross-β sheet conformation [1]. The assembly of normally soluble proteins into amyloid fibrils is often associated with devastating neurological disease. To date, approximately 50 human diseases have been linked to the formation of amyloids, including Alzheimer’s (AD), Parkinson’s (PD) and Huntington’s (HD) diseases, and transmissible spongiform encephalopathies (TSEs), or prion diseases [2]. Prions are self-perpetuating protein isoforms, usually of amyloid nature that are transmitted via extracellular infection in mammals. This capacity to seed, or template, the conversion of respective soluble protein into an aggregated pathogenic form is the basis of prion infectivity.

However, the ability to form self-templating amyloid is not unique to proteins traditionally designated as prions. There is a growing understanding that the more common neurodegenerative diseases, including AD and PD, spread in brains by a mechanism somewhat analogous to prion transmission [3,4,5,6,7]. Several neurodegenerative diseases are associated with the accumulation of self-templating amyloid forms of specific proteins, such as β-amyloid (Aβ) and tau in AD, α-synuclein in PD, and huntingtin in HD. Typically, amyloidogenesis is a specific self-seeding process in which the amyloid form of a protein only converts other copies of the same protein and not proteins with a different primary sequence. However, on rare occasions, so-called ‘cross-seeding’ occurs, when an amyloid form of one protein catalyzes the assembly of another protein into an amyloid. Usually, cross-seeding is not as effective as self-seeding, but it may play an important role in the initiation of fiber assembly from a non-amyloid state. Cross-seeding events might also have an important role in neurodegenerative disorders. For example, pure α-synuclein and tau synergize to promote the fibrillization of each other [8]. Recent evidence of prion-like propagation of several misfolded proteins from cell to cell within the brains, if not from tissue to tissue, raise concerns that various protein misfolding diseases might have spreading, prion-like etiologies that contribute to pathogenesis.

Considering the high incidence of AD, PD and HD, it is crucial to understand if some cases can be initiated by transmission events. Although there is little or no evidence of human-to-human transmission of these diseases, prion properties of respective proteins were uncovered in the cellular or animal models [9,10], and as protein misfolding within individuals apparently propagate via a prion-like mechanism, it is important to know how it can be altered to change a course of disease. The search for therapeutic treatments against amyloid/prion diseases spans more than 30 years, but has had only limited success [1,11].

## 2. Yeast Prions and Protein Quality Control

Regardless of its relative simplicity, yeast harbors a significant number of cellular pathways and factors relevant to human neurodegeneration, including conserved chaperone and protein remodeling, the ubiquitin proteasome system, secretion, vesicular trafficking, and autophagy. The high degree of conservation enables researchers to reliably model disease mechanisms in a highly controllable environment.

Yeast prions are endogenous heritable amyloids, most often studied in the yeast *Saccharomyces cerevisiae* [12,13,14,15]. The molecular foundation of inheritance for yeast prions and mammalian amyloids is through nucleated polymerization of amyloid fibrils. The phenotypic effects of prion formation are typically manifested as a decrease of protein function in the amyloid state. Due to convenient genetic and phenotypic assays, yeast prions provide a useful model system for studying mechanisms of amyloid formation and propagation that are mostly applicable to mammalian and human diseases [12,13,14,15].

**Figure 1:**
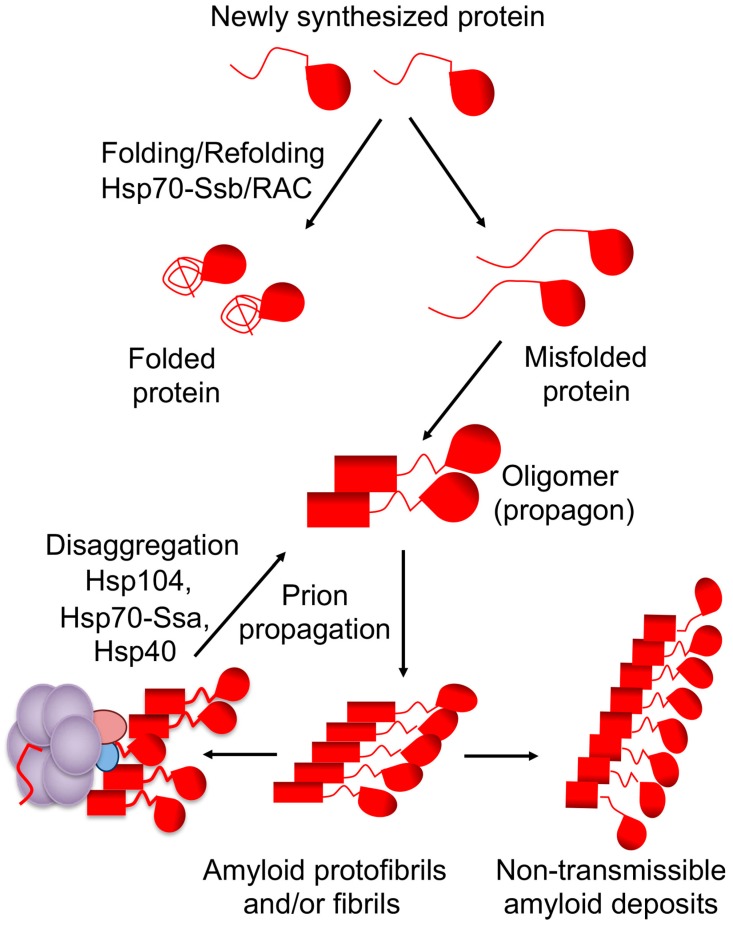
”Life cycle” of the [*PSI^+^*] prion and the role of chaperone machinery. Chaperone Hsp70-Ssb with the cochaperones of the ribosome-associated complex (RAC) assist in normal protein folding, thus counteracting misfolding. Misfolded proteins assemble into amyloidogenic oligomers, producing amyloid fibrils. In the case of a prion, amyloids are fragmented by chaperone complex Hsp104/Hsp70-Ssa/Hsp40 into oligomeric “propagons” (transmissible amyloids), continuing the propagation cycle after cell division. Non-fragmented fibrils generate large non-transmissible amyloid deposits, which do not re-enter the propagation cycle and/or are malpartitioned in cell divisions.

Cellular defense machineries such as chaperone proteins and the ubiquitin–proteasome system, aimed at protecting the cells from aggregation of stress-damaged proteins, also recognize amyloid aggregates and stress-related proteins and serve as major modulators of prion formation and propagation in yeast [16,17,18,19,20,21]. The same chaperone machinery that is involved in disaggregation of stress-damaged proteins is involved in propagation and inheritance of yeast prions [17,18]. The connection between chaperones and prions was first established using [*PSI*^+^] prion as a model (Figure 1). The chaperone machinery fragments large fibrils into small oligomers, which initiate a new cycle of prion replication. The first identified component of this machinery is a chaperone protein Hsp104 [22]. The Hsp104 is essential for all amyloid-based cytosolic yeast prions [18,23]. The machinery also includes members of the Hsp70 family Ssa [24,25,26,27] and cochaperones of the Hsp40 family, also known as J-proteins [28,29,30]. In the current model of [*PSI^+^*] prion propagation, the Hsp70/40 complex binds to amyloid fibrils and recruits Hsp104 [31]. While Hsp104 is required for prion propagation, overproduction of Hsp104 destabilizes or “cures” some yeast prions, for example [*PSI^+^*] and [*MOD^+^*], and at high levels, [*URE3*] [17,18,22,23,32]. Potentially this anti-prion effect is due to the fact that direct binding of Hsp104 to amyloid fibrils (without Hsp70-Ssa) is not only incapable of fragmenting fibrils but also antagonizes prion propagation [31]. Proposed (and mutually non-exclusive) models for prion curing by excess Hsp104 include removal of monomers from the termini of fibrils, resulting in eventual destruction of prion polymers [33], and prion mal-partition during cell divisions [13,34] (see recent experimental evidence [35]).

Although most components of the chaperone machinery are evolutionarily conserved, Hsp104 orthologs are not present in the cytosol of multicellular animals, including mammals. At the same time, it was demonstrated that the chaperone system Hsp70-Hsp40-Hsp110 can promote protein disaggregation in mammalian cells [36,37,38,39,40]. To date, auxillary proteins involved in propagation of mammalian amyloids remain to be identified. Recent data [41] suggest that some Hsp104 functions could be assumed by its distant mammalian paralogs, RuvbL1 and RuvbL2, whose orthologs are also present in yeast under the names of Rvb1 and Rvb2, respectively. However, the impact of RuvbL1/2 on prions still needs to be investigated. At the same time, potentiated variants of Hsp104 have been engineered to disaggregate misfolded proteins of higher eukaryotes, connected with PD (α-synuclein) and amyotrophic lateral sclerosis (ALS) (TDP-43 and FUS) [42,43]. Using lessons learned from yeast models, similar potentiated human protein disaggregases, such as Hsp110/Hsp70/Hsp40 [40] and HtrA1 [44], could be engineered. Another approach is identification of small-molecule enhancers of the chaperone activity, that could potentially yield transformative therapeutics for ALS, PD, and AD [43]. Potential danger, associated with these approaches, is that as we saw in yeast, modulations of chaperone activity may work in both directions, for example, increased chaperone activity may in fact promote amyloid propagation through increased fragmentation. Further understanding of the mammalian chaperone machinery, associated with amyloids, is necessary for successful development in this direction.

## 3. Contribution of Environmental Factors to Amyloid Disease

It is widely believed that environmental exposures contribute to the vast majority of sporadic Alzheimer’s, Parkinson’s, Huntington’s and prion diseases alone or via interactions with genetic factors [45,46,47,48,49]. Epidemiological studies have associated environmentally persistent organic pollutant exposure to brain disorders [46]. Proven and potential neurotoxic substances include heavy metals, organic solvents, persistent organic pollutants, plastic exudates, pesticides, brominated flame retardants, and polycyclic aromatic hydrocarbons [45,50,51,52,53,54]. Smoking is implicated in a decreased risk of developing Parkinson’s disease [45,53] and caffeinated coffee consumption is associated with a reduced risk of PD and AD [55], but this association is controversial. According to recent discoveries, the PD patients are less likely to establish smoking habits, because of a decreased responsiveness to nicotine and that ease of smoking cessation is an early manifestation of premotor PD related to the loss of nicotinic rewards [56]. This should be noted that effects of environmental risk factors identified thus far are characterized only for specific amyloid diseases, so that it is not clear if any of them have a general pro-amyloid effect.

With the proliferation of electric devices and wireless communication equipment, the concern was raised about the health effects of extremely low-frequency electromagnetic field (ELF-EMF) and radio frequency electromagnetic field (RF-EMF). It was found that exposure to ELF-EMF could increase production of amyloid beta (Abeta), an amyloidogenic protein associated with AD, and elevate the risk of AD [57]. At the same time, exposure to RF-EMF has some beneficial effects in regard to AD pathology in a transgenic model [58,59], and its beneficial effect was also reported from the epidemiological survey of AD and PD patients [60].

Aging is the primary non-genetic risk factor for sporadic AD. The early-life environment was implicated as one of primary factors in defining an individual’s susceptibility to AD and PD [61,62,63]. Fundamental aging-related processes, such as decreased adaptation to stress and accumulation of reactive oxygen species (ROS), as well as a decline in protein homeostasis, may serve as initiators of Aβ and prion aggregation [64,65,66]. The current model of AD considers amyloid formation by Aβ as a triggering factor in AD [67].

Various environmental stresses may impact amyloids and prions via different mechanisms; therefore, studying the environmental triggers and modifiers of neurodegenerative diseases is critically important. In contrast to genetic factors, environmental factors potentially could be modified, and this may have a dramatic effect on prevention, occurrence and treatment [45]. Yeast model systems described below provide an excellent tool for the investigation of the impact of environment on the formation and propagation of amyloids.

## 4. Effects of Chemical Agents and Environmental Factors on the Formation of Yeast Prions

Molecular mechanisms triggering conversion from a normally soluble protein into the amyloid/prion form remain largely unknown. Understanding these mechanisms is central to the development of both prophylactic recommendations and effective therapeutic strategies, aimed at amyloid diseases. Yeast models provided important data showing how amyloids and prions arise in vivo. For example, transient overproduction of a prionogenic protein results in prion formation [68,69,70]. Once prion assemblies are generated, they can be propagated even at normal expression levels of a prionogenic protein [18]. This process is greatly facilitated by the presence of other proteins in an aggregated state, suggesting that cross-seeding interactions can nucleate de novo amyloid/prion formation in yeast [71,72].

A variety of environmental stress conditions are known to increase the frequency of prion formation in yeast. This is in an agreement with the fact that conditions favoring protein misfolding may also favor the conversion of a normally soluble protein into an amyloid form [17,73]. For example, formation of the yeast prion [*PSI^+^*], an aggregated form of the translation termination factor Sup35, is facilitated by prolonged incubation at low temperature [16], heat stress [74], osmotic and oxidative stresses [75,76], the unfolded protein response and ER stress [34,73,77]. Typically, these effects are detected in the strains containing another protein, such as Rnq1, in an amyloid form. Rnq1 prion is known to increase [*PSI^+^*] formation, possibly via a cross-seeding mechanism [71,72,78].

De novo generation and propagation of another yeast prion [URE3] increased after the exposure to low-frequency (ELF-EMF) and radio-frequency (RF-EMF) electromagnetic fields [79]. The observation that production of ROS, as well as the activities of superoxide dismutase (SOD) and catalase (CAT), but not the levels of chaperone proteins, were elevated in yeast cells in these conditions supports the hypothesis that ROS may play a role in the effects of EMF on protein misfolding and amyloid formation.

**Figure 2:**
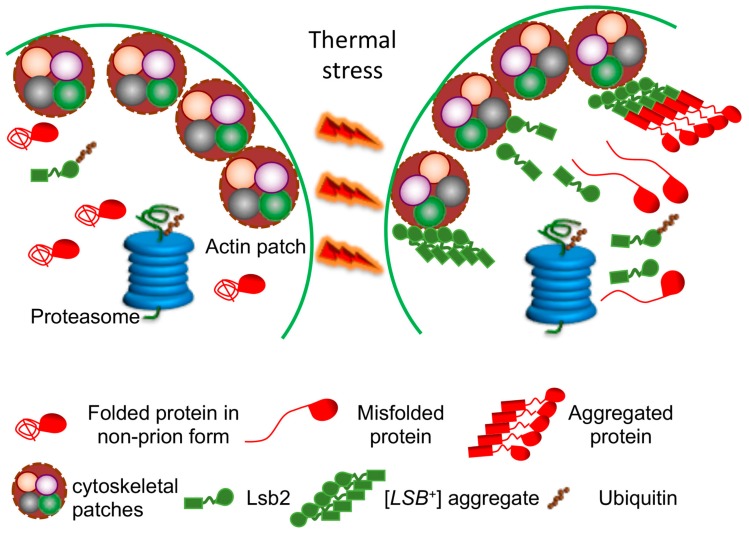
Lsb2 aggregation and prion formation during thermal stress. Thermal stress (39 °C) leads to an increased synthesis of the Lsb2 protein, as well as to misfolding of other proteins. When present at high concentration, Lsb2 forms prion-like aggregates ([*LSB*^+^]), which are associated with peripheral cytoskeletal patches and promote assembly of misfolded proteins into protective (but potentially amyloidogenic) aggregate deposits. [*LSB*^+^] aggregates are metastable and lost in cell divisions after stress, while the Lsb2 protein is ubiquitinated and degraded by a proteasome.

Active adaptation of yeast cells to environmental stress apparently involves conversion of some normally soluble proteins into an aggregated (and in some cases, amyloid) form. It is possible that amyloid formation may promote survival under stress conditions, for example, by assembling the damaged proteins into amyloid deposits. Thus, minimizing their damaging effect to the cell. Reversible assemblies may also help to protect essential proteins from degradation machinery, activated during stress, as proposed in [16]. Our data show that the yeast stress-inducible cytoskeleton-associated protein, Lsb2, forms a metastable prion [*LSB^+^*] in response to high-temperature stress [80]. This prion has been shown to promote conversion of other cellular proteins into a prion form [80,81] (Figure 2). These data demonstrate a possible role for Lsb2 as a sensor of stress. Apparently, Lsb2 acts as a transient catalyst of heterologous prion formation due to its ability to form a transient stress-inducible prion state that facilitates the potentially cytoprotective assembly of other aggregation-prone proteins into deposits at specific cytoskeleton-associated sites [81]. The metastable stress-inducible Lsb2 prion confers the memory of stress to a subpopulation of yeast cells. If the prion form of Lsb2 is playing an adaptive role, such a stress memory could be adaptive during repetitive stresses, via conferring increased stress “awareness” and, therefore, increased stress resistance to the prion-containing cells. Notably, the ability of Lsb2 to form an aggregated state and to promote aggregation of other proteins is confined to a single amino acid substitution which has been acquired in evolution at the same time when Saccharomyces yeast adapted to higher growth temperatures. Therefore, it is possible that prion-based stress memory has arisen as a defensive tool intended to minimize the pathogenic effects of the increased accumulation of misfolded proteins and to prevent degradation of essential proteins under unfavorable conditions [16,82].

Another environmentally regulated and potentially adaptive yeast prion is [*MOT3^+^* ] [83]. It is a prion form of transcriptional factor Mot3, which regulates genes involved in cell wall and ergosterol biosynthesis in yeast. Mot3 is also involved in repression of anaerobic genes during aerobic growth and reduction in Mot3 levels occurring in hypoxic cells results in the de-repression of the target anaerobic genes. Formation, elimination and phenotypic manifestation of the [*MOT3^+^*] prion are all modulated by specific environmental conditions. Formation of the [*MOT3^+^*] prion results in the acquisition of an adhesive phenotype, formation of multicellular chains and generation of a more elaborate biofilm. Ethanol stress increases the frequency of [*MOT3^+^*] formation, while hypoxia eliminates [*MOT3^+^*], possibly due to a decrease in Mot3 protein levels. In natural conditions, yeast cultures frequently undergo transitions from high ethanol stress (caused by utilization of sugars via brewing) to hypoxia. Thus, formation and loss of [*MOT3^+^*] prion might work as a molecular switch that occurs sequentially in the natural fermentation/respiration cycles of yeast populations and contribute to the natural morphological diversity of budding yeast [83].

Formation of the [*MOD^+^*] prion, by a tRNA modification enzyme, Mod5, was observed when non-prion yeast was grown under selective pressures from antifungal drugs [84]. [*MOD^+^*] cells accumulate more ergosterol and are resistant to ergosterol synthesis inhibitors, such as fluconazole and ketoconazole—common antifungal drugs. However, it remains uncertain if [*MOD^+^*] is induced by azoles or simply selected in their presence. Connections to some drugs are also described for other yeast prions. For instance, the prion form of a chromatin remodeler, Swi1 [85], leads to formation of the prion state [SWI*^+^*], which is resistant to microtubule disrupting drugs [86]. Similarly, the antibiotic G418 increases the frequency of [URE3] prion induction [87]. In this case, [URE3] prion does not confer the resistance to an antibiotic, instead the antibiotic treatment increases the rate of translational errors, which apparently results in an increase of the frequency of Ure2 misfolding and prion formation.

An interesting example of the environmentally regulated prion is [*GAR^+^*], a membrane-associated heteromeric complex consisting of the plasma membrane proton pump Pma1 and the glucose-repressed gene regulator Std1. In contrast to most other yeast prions, it is not proven that [*GAR^+^*] is associated with an amyloid state. Also, it appears that [*GAR^+^*] generation involves some changes in the protein complex assembly. Formation of [*GAR^+^*] occurs with nutrient fluctuations in the environmental niche and reverses glucose-associated repression in *S. cerevisiae* [88,89]. Notably, [*GAR^+^*] is induced across an entire population in response to lactic acid secreted by certain bacterial species [90,91] and eliminated by desiccation [92]. As [*GAR^+^*] cells produce less ethanol and, therefore, do not inhibit growth of bacteria, [*GAR^+^*] induction is certainly beneficial to bacterial cells producing the [*GAR^+^*]-inducing compounds. It was argued that [*GAR^+^*] could also be beneficial to yeast due to an increased choice of utilized carbon sources. This could be true in a general sense, although it is not clear if induction of [*GAR^+^*] is beneficial to yeast in the particular situation of the mixed yeast/bacterial community.

## 5. Clearance of Yeast Prions by Chemical Agents and Environmental Factors

In vivo clearance pathways for misfolded proteins include the ubiquitin–proteasome system (UPS) and the autophagy–lysosome network (ALN) [93,94]. Some data connect these pathways to clearance of amyloid aggregates, although effects are not straightforward. For example, proteasomes are not likely to be efficient in degrading aggregated proteins, although they may counteract subsequent aggregation by degrading misfolded precursors. One of the approaches to aggregate clearance in proliferating cells is asymmetric segregation in cell divisions [95]. Chaperone proteins participate in all these pathways and make a significant impact on amyloid clearance.

Incubation with various chemical agents, such as guanidine hydrochloride (GuHCl), dimethylsulfoxide, ethanol, methanol, glycerol, succinate, glutamate and MgCl_2_ ”cures” yeast cells of some prions [73,96]. The mechanism behind action of these chemicals is largely unknown, with the exception of GuHCl, which is an inhibitor of Hsp104 [97,98,99,100], a major chaperone required for yeast prion propagation (see ref. [22] and above, Figure 1). Growth of yeast cultures in the presence of millimolar concentrations of GuHCl cures most of yeast prions known to date in a generation-dependent manner, due to a defect in fibril fragmentation and production of new seeds, so that pre-existing prion units are diluted and, eventually, lost upon cell division. This should be noted that some other abovementioned anti-prion agents influence levels of yeast Hsps, thus it is possible that they also act via a modulation of the chaperone machinery.

Some environmental stresses, such as severe heat shock, also cause loss of the [*PSI^+^*] prion [96], although a mild increase in growth temperature was initially reported to have no effect. However, it was then shown that short-term exposure of exponentially growing yeast culture to mild heat shock (e.g., 39 °C), followed by immediate resumption of growth, leads to destabilization of the [*PSI^+^*] prion, that is most pronounced in so-called “weak” prion variants [34]. (Variants, or “strains” of prion likely represent amyloid isoforms with different structures of a cross-β core region, see refs. [13,101]). Most of prion destabilization occurs due to impairment of prion segregation in the divisions following resumption of cell proliferation [34,102]. Longer incubation at increased temperature results in prion recovery. Remarkably, both prion destabilization and recovery depend on protein synthesis, and maximal prion destabilization coincides with maximal imbalance between Hsp104 and other Hsps, such as Hsp70-Ssa [19,34]. This is consistent with the notion that efficient prion fragmentation and segregation requires a proper balance between Hsp104 and Hsp70-Ssa chaperones. Segregational prion loss after heat shock was attributed to either malpartition of prion aggregates under conditions where their normal proliferation is impaired due to altered Hsp balance [34], or asymmetric distribution of excess Hsp104 in cell divisions following heat shock [103]. These explanations are not mutually exclusive. Recent data [104] show that [*PSI^+^*] destabilization by mild heat shock is significantly decreased in the absence of protein deacetylase Sir2, previously implicated in the control of asymmetric segregation of the aggregated heat-damaged proteins in the cell divisions following heat shock [105]. Indeed, the aggregates of Sup35 tagged with Red Fluorescent Protein (RFP) colocalize with the Hsp104 (a marker of the deposits of heat-damaged proteins) tagged with Green Fluorescent Protein (GFP) in heat shocked cells and show a tendency of mother-specific accumulation in the post-heat-shock cell divisions [104]. Notably, the abovementioned cytoskeleton-associated stress-inducible prionogenic protein, Lsb2, and its non-prionogenic paralog, Lsb1, partially protect [*PSI^+^*] from destabilization by mild heat shock, consistent with their general “pro-aggregation” effect [81,102]. Another prion eliminated by growth at a mildly elevated temperature is [*SWI^+^*] [27], although the detailed mechanism of curing has not been deciphered in this case.

Osmotic stress also causes loss of the [*PSI^+^*] prion [34,96]. However, in contrast to heat shock, [*PSI^+^*] destabilization by osmotic stressors does not necessarily depend on cell proliferation and/or protein synthesis [34], indicating that different stresses may impact the prion via different mechanisms.

Nutrient deprivation (that is, growth in poor synthetic medium) results in the increased loss of some variants of the [*PSI^+^*] prion [106]. This was attributed to an increased release of chaperone Hsp70-Ssb from the ribosome-associated complex (RAC) into cytosol. Indeed, RAC disruption due to depletion of Hsp40-Zuo1 or Hsp70-Ssz1 (cochaperones, composing the ribosome-associated complex, RAC that links Hsp70-Ssb to translating ribosomes) also has a destabilizing effect on [*PSI^+^*] propagation. An excess of Hsp70-Ssb in the cytosol antagonizes binding of another Hsp70 chaperone, Ssa to prion aggregates, that impairs prion propagation [106,107]. Release of Hsp70-Ssb from the ribosome is also detected during heat shock, and both single deletions of either of the genes coding for Hsp70-Ssb, *SSB1* or *SSB2*, or double deletion of both genes (*ssb1/2Δ*) decrease destabilization of [*PSI^+^*] by mild heat shock [104]. In contrast, deletion of either gene coding for the RAC component, *zuo1Δ* or *ssz1Δ*, increases [*PSI^+^*] destabilization by heat shock. This effect of RAC disruption on [*PSI^+^*] is, in a significant part, mediated by Hsp70-Ssb, as it is ameliorated in the triple *ssb1/2Δ zuo1Δ* strain [104]. These data show that intracellular relocalization of the heat shock non-inducible chaperone, Hsp70-Ssb, modulates propagation of protein aggregates after heat shock. Possibly, Hsp70-Ssb released from the ribosome into cytosol antagonizes Hsp70-Ssa, thus further increasing the imbalance between Hsp104 and Hsp70-ssa proteins, bound to prion aggregates. Both orthologs of RAC components and ribosome-associated Hsp70s that are functionally analogous to Hsp70-Ssb are found in human cells [108]. This makes it likely that RAC-dependent regulation of amyloid aggregation is not restricted only to yeast [107].

Alterations in protein degradation pathways have been linked to both heritable and sporadic aggregation-related neurodegenerative diseases [109]. Protein ubiquitination is a reversible post-translational modification in which the 76 aa polypeptide called ubiquitin (Ub) is covalently linked, via its C-terminal glycine residue to the ε-amino group of lysine residues in target proteins [110]. UPS failure leads to the accumulation and aggregation of misfolded proteins [93,111], which may result in enhanced nucleation of amyloids. On the other side, accumulation of protein aggregates can sequester Ub and other UPS components, inhibiting the proteasome and exerting pleiotropic effects on cellular metabolism in target proteins. UPS defects have been linked to certain amyloid and neural inclusion diseases in mammals and humans [112]. In yeast, UPS alterations influence formation and propagation of the [*PSI^+^*] prion [20,21]. De novo [*PSI^+^*] induction by excess Sup35 is more efficient at increased Ub levels, and is reduced by a decrease in the levels of free Ub, for example, in the strains lacking the deubiquitinating enzyme Ubp6 [20]. Deletion of *UBC4*, which encodes one of the major yeast ubiquitin conjugating (E2) enzymes, increases both [*PSI^+^*] resistance to “curing” by overexpressed chaperone Hsp104 and de novo [*PSI^+^*] formation [21]. The simplest explanation for the effect of *ubc4Δ* (and possibly, other UPS-deficient deletions) on [*PSI^+^*] would be that a defect in ubiquitination prevents degradation of misfolded Sup35, thereby increasing its abundance and conversion into a prion. However, despite numerous searches, there is no evidence for direct ubiquitination of Sup35. Another (not mutually exclusive) explanation could be that *ubc4Δ* acts via auxillary factors. Indeed, the amount of the Hsp70-Ssa chaperone associated with Sup35 aggregates is increased in the *ubc4Δ* cells [21]. Hsp70-Ssa is known to promote the formation and propagation of [*PSI^+^*] (see above), and is itself ubiquitinated [113]. Yeast cytoskeletal protein Lsb2 that triggers [*PSI^+^*] prion formation and protects [*PSI^+^*] from destabilization during stress ([81,102], see above) is ubiquitinated and degraded via the proteasome [81]. The metastable nature of the [*LSB^+^*] prion could be at least partly related to the proteolytic instability of its carrier protein because mutations impairing ubiquitination and subsequent degradation of Lsb2 also increase transmission of [*LSB*^+^] in cell divisions [80].

Autophagy is a non-selective degradation process which destroys the bulk of cytoplasm and/or whole organelles and recycles macromolecules in response to starvation conditions. Autophagy can serve as a protein quality control mechanism degrading protein aggregates [114]. Spermidine, a polyamine that has been used to induce autophagy, has been reported to “cure” yeast cells of the prion forms of proteins Sup35 ([*PSI^+^*]) and Rnq1 ([*PIN^+^*]) [76,115].

## 6. Yeast Models for Discovery of Anti-Prion Drugs

*S. cerevisiae* yeast has been successfully used to model protein aggregation in human disorders including AD, PD, HD and TSEs. The low cost of yeast experiments and the availability of high-throughput techniques makes yeast suitable for large-scale genetic and pharmacological screens. More than 1000 genes involved in human disorders have orthologs in the yeast genome. These genes can be genetically and functionally replaced by their human equivalents. The creation of “humanized” yeast strains with whole pathways modified to resemble human cell biology [116,117] facilitates the use of yeast in studying human diseases. Yeast has become a widely used tool for discovery of new drugs and their mechanisms of action, and this has been applied to amyloids and prions as well.

**Figure 3:**
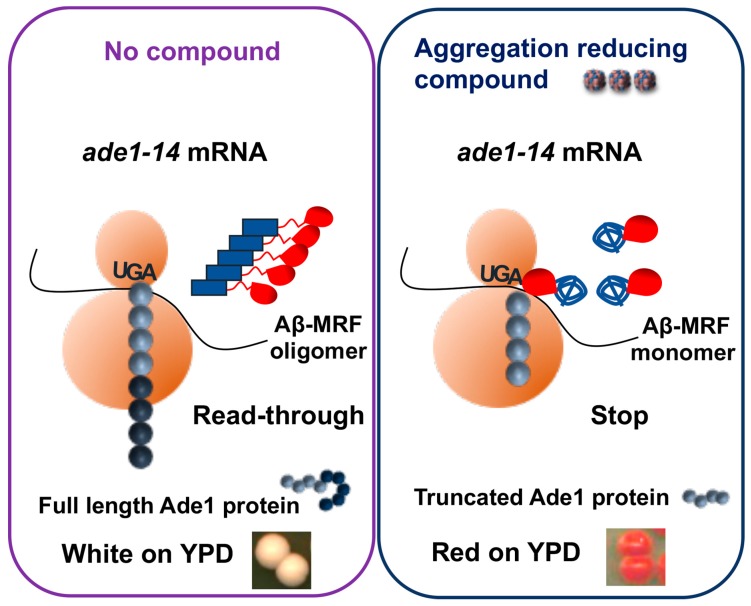
Yeast model to screen for inhibitors of Aβ oligomerization. In the *ade1-14* reporter strain, the stop codon UGA, introduced into the *ADE1* gene, is normally recognized by the translation termination complex, including release factor Sup35. Fusion of Aβ with functional domain (MRF) of Sup35 leads to its oligomerization. When Aβ-MRF is in an oligomeric for, translation termination is impaired. This results in synthesis of full-length Ade1 protein due to readthrough of the stop codon, inability of cells to grow on the medium lacking adenine (–Ade) and white color on the complete (YPD) medium (Left panel). If cells are treated with a compound able to counteract oligomerization of Aβ-MRF, translation termination is restored, leading to the production of truncated Ade1 protein, inability of cells to grow on -Ade medium and accumulation of red pigment (a polymerized intermediate of the adenine biosynthetic pathway) on YPD medium (right panel).

A red-/white colorimetric assay for identification of antiprion compounds (Figure 3) has been developed on the basis of the yeast prion [*PSI^+^*]. [*PSI^+^*] is an aggregated, partially inactive isoform of translation termination factor Sup35. Therefore, readthrough of stop codons occurs in the cells bearing [*PSI^+^*]. The detection assay employs a specifically designed yeast strain containing a stop codon (nonsense-mutation) in the middle of the coding region of *ADE1* gene [13]. When Sup35 is in an active soluble form, it terminates translation at the stop codon. As a result, yeast cells cannot grow on a metabolic medium lacking adenine and accumulate a red pigment generated by an intermediate. When Sup35 is present in its aggregated prion form, it fails to terminate translation and the ribosome reads through the nonsense codon. This allows cells to grow on the medium lacking adenine and cells growing on rich medium are white, because accumulation of the red intermediate is prevented. This assay was used for safe and high-throughput screening of anti-prion compounds. To increase sensitivity, an anti-prion compound, GuHCl, was added to the yeast medium at a low concentration. A chemically diverse library of 2500 compounds (synthetic and natural products purified from various sources by academic laboratories) was screened for the ability to cure the [*PSI^+^*] phenotype, detected by the generation of a red halo surrounding a disk of filter paper with a tested compound on a Petri dish [118,119]. [*PSI^+^*]-curing compounds were then tested for their activity against another yeast prion, [*URE3*] followed by the analysis of their effects on the pathogenic mammalian prion protein PrP^Sc^ (associated with TSEs) in a cell-based assay and mouse models [120]. Notably, quinacrine and chlorpromazine, shown to promote mammalian PrP^Sc^ clearance in cell cultures, were also active in the yeast-based method. Imiquimod (IQ), a potent Toll-like receptor 7 agonist, imiquimod, was identified as new compound with anti-prion activity against yeast prion [*PSI^+^*] and [*URE3*] [121]. IQ also has anti-prion activity against mammalian prions and was already in clinical use. Biochemical and genetic studies reveal that IQ and two other compounds identified in yeast assay, 6-aminophenanthridine and guanabenz acetate, target ribosomal RNA (rRNA) and specifically inhibit the protein folding activity of the ribosome (PFAR) [122], borne by domain V of the large subunit rRNA. PFAR is evolutionarily conserved and could be a potential therapeutic target for human protein misfolding diseases [123].

The same principle was used for an optimized liquid phase micro-culture assay, operating with yeast strains, carrying prions [*PSI^+^*] and [URE] [124] and applied to identification of natural inhibitors of yeast prions in extracts of marine invertebrates, collected from temperate waters in Australia. As a result, several bromotyrosine derivatives from the extract of *Suberea ianthelliformis* were identified as potent inhibitors of yeast prions. All anti-prion compounds from the sponge extracts contained an ethylaminodibromophenyl (EADP) moiety. This may serve as a useful lead for the future development and design of novel and improved anti-prion therapeutics [124].

## 7. Yeast Models for Identifying Candidate Drugs Against Alzheimer’s Disease

Yeast models are also playing an increasingly important role in unravelling the fundamental disease aspects of AD [125]. The triggering event in Alzheimer’s disease is believed to be the aggregation of the β-amyloid (Aβ) peptides [67]. The predominant types of Aβ peptide in human cells are Aβ40 and Aβ42, of which the latter one is considered to be most aggregation-prone and pathogenic. Extensive evidence suggests that the primary neurotoxic effects are associated with smaller (dimers, trimers, and tetramers) oligomers of Aβ42, which seem to appear during the early stages of Aβ42 assembly [126]. Various yeast models for studying Aβ aggregation by screening chemical compounds that reduce Aβ aggregation or oligomerization were developed [127,128]. In one model, Aβ42 was substituted for the prion domain of yeast translational termination factor Sup35 (Aβ42-MRF). The functional region of Sup35 was retained as a reporter, allowing for a red/white assay based on the same principle as described above (Figure 3). The fact that yeast ortholog of the human AD risk factor, phosphatidylinositol-binding clathrin assembly protein (PICALM), reduces oligomerization of Aβ42-MRF indicates that Aβ42-MRF reporter system is suitable for identifying compounds that could be developed into therapies that prevent or arrest AD [129]. This approach was used to screen for agents that reduce abundance of Aβ42 oligomers [127,130]. Two presumptive anti-oligomeric compounds were identified from a sub-library of 12,800 drug-like small molecules [131], and seven compounds were identified from a screen of 1200 FDA-approved drugs and drug-like small molecules [129]. These include: three antipsychotics (bromperidol, haloperidol and azaperone), two anesthetics (pramoxine-HCl and dyclonine-HCl), tamoxifen citrate, and minocycline-HCl. All seven drugs caused Aβ42 to be less toxic to cultured PC12 human cells. One potential disadvantage of this assay is that the chimeric construct oligomerizes instantly in yeast cells, thus it is possible to look for agents counteracting existing oligomers but may not be as useful for those acting at initial oligomer nucleation.

Several labs employed Aβ40 or Aβ42, fused to fluorescent protein, green (GFP), yellow (YFP) or cyan (CFP), and expressed in yeast. The fusion protein spontaneously misfolds and aggregates. Depending on the type of construct, this either allowed for microscopic detection of Aβ-based aggregates in yeast [132], or suppressed green fluorescence [128,133]. Suppression of fluorescence in the GFP-Aβ constructs was used to screen for compounds that increase fluorescence, with the hope that such compounds would antagonize aggregation and play a protective role against AD. Folinic acid was uncovered from such a screen, suggesting folate can assist with preventing Aβ-misfolding/aggregation [128].

Fluorescently detected Aβ-based aggregates were shown to interact with mammalian PrP protein in yeast cells [132]. This reproduces results previously described for mammalian and human cells [134]. The Aβ-PrP interaction was shown to play a role in AD pathology [135], although its specific impact is still unclear.

The GFP-Aβ model was also used to test rationally designed compounds with the potential anti-amyloid effect [136]. The hydrophobic core region encompassing residues 11 through 25 is thought to be crucial for Aβ assembly into fibrils, and peptides representing portions of this region can bind full-length Aβ. Of those, pentapeptides KLVFF or LVFFA were used as recognition units in the design of inhibitors of Aβ fibrillization. Such peptidomimetics showed moderate to good activity in both inhibition and dissolution of Aβ aggregates as demonstrated by thioflavin assay, circular dichroism measurements and microscopy. They also ameliorated the toxicity caused by GFP-Aβ in yeast and were able to clear the GFP-Ab aggregates in vivo in an autophagy-dependent manner [136].

Another anti-histamine drug, Latrepirdine (Dimebon™), which has shown some benefits in trials of Alzheimer disease [137], was demonstrated to reduce levels of GFP-Aβ42 aggregates and attenuated Aβ42-induced toxicity in yeast [138]. In the yeast AD model, Latrepirdine upregulates yeast vacuolar (lysosomal) activity and promotes transport of the autophagic marker (Atg8) to the vacuole. The mechanism of Latrepirdine action in the clearance of Aβ42 aggregates via induction of autophagy was later confirmed by a mouse AD model [139].

In all the abovementioned studies, Aβ was expressed in the cytoplasm of yeast cells. To recapitulate the Aβ secretion and endocytosis observed in human brains, a new yeast model was developed that is based on a secreted form of Aβ [140,141]. For this purpose, Aβ42 was fused to either the endoplasmic reticulum-targeting signal (ssAβ42-GFP) [140], or the mating factor α (MFα) signal peptide (MFα- Aβ42-GFP) [141], so that multi-compartmental distribution of Aβ42 was successfully mimicked in yeast. Expression of ssAβ42-GFP disrupted normal cellular endocytic trafficking (possibly due to accumulation of Aβ in the space outside of the cell membrane), which results in cytotoxicity [140]. Over 140,000 compounds were screened for the reversal of toxicity, and a class of protective metal-binding compounds related to clioquinol (CQ), a compound that alleviates Aβ toxicity in mouse AD models was identified [140]. These structurally dissimilar compounds strongly synergized at concentrations otherwise not competent to reduce toxicity. They were able to increase Aβ turnover, restore vesicle trafficking and provide oxidative stress protection. Treatment with clioquinol-related compounds inhibited Aβ accumulation and resulted in a dramatic improvement in learning and memory in mouse transgenic models [142] and human patient cohorts [143]. Notably, drugs identified in the yeast screen for antagonists of oligomerization [129] were also active in the toxicity assay [144]. The major disadvantage of this assay is that it is aimed at Aβ42 accumulation and secretion, rather than at aggregation or oligomerization per se.

## 8. A Yeast Model for Discovery of Drugs against Huntington’s Disease

The budding yeast *Saccharomyces cerevisiae* has recently emerged as an effective tool to study Huntington’s disease (HD) [145]. A hallmark of HD is the accumulation of aggregates of huntingtin protein (Htt) or its N-terminal fragment containing the polyQ repeat [146]. A poly(Q)-length-dependent model of Htt aggregation was established by fusing the first 68 N-terminal amino acids of wild-type HTT exon-1 containing poly(Q) tracts of varying length (25, 42, 72 or 103 glutamines) with a C-terminal GFP (green fluorescent protein) tag [147,148]. Aggregation of Htt-GFP in yeast depends on the length of the polyQ repeat, so that polyQ expansion promotes aggregation as in humans. PolyQ-dependent aggregation is toxic to yeast cells and can be modified both by genetic and pharmacologic means. Some yeast [149] or mammalian [150] chaperones of the Hsp40 family were shown to counteract aggregation and toxicity of the Htt-based polyQ constructs in the yeast model, agreeing with data obtained in mammalian models [151]. Notably, aggregation and toxicity of the Htt exon-1 based polyQ constructs in yeast cells is promoted by the presence of the endogenous yeast QN-rich prions, such as Rnq1 [148]. In contrast, the presence of the P-rich sequence, which immediately follows the polyQ stretch within exon-1 of Htt, ameliorates cytotoxicity by facilitating the assembly of polyQ aggregates into a protective aggregate deposit, reminiscent of the mammalian aggresome [119]. Still, the aggresome becomes toxic in the presence of [*PSI^+^*], prion form of the translation termination factor Sup35 (eRF3), as the aggregated form of Sup35 mediates sequestration of another translation termination factor, Sup45(eRF1), by polyQ aggregates [152]. These data show that the composition of endogenous aggregated proteins serves as a major modulator or Htt aggregation and toxicity at least in yeast (and possibly in humans). Therefore, both the prion composition of the reporter yeast [153] and Htt-based polyQ constructs in yeast are important.

Several types of cellular dysfunction that are observed in HD patients and higher eukaryote HD models are also found in HD yeast models. These include impairment of endocytosis [154,155], dysfunction of mitochondria [156], increased levels of ROS [157], dysregulation of transcription [158], induction of apoptotic markers [153]. Yeast models of HD have been successfully used to identify new potent compounds with therapeutic potential. A yeast-based approach based on the aggregation and cytotoxicity of Htt-103Q–GFP was used to screen a library of 16,000 small chemical compounds [159]. Effects of the newly identified compounds were further validated in mammalian cell-based models of HD, and in the transgenic mouse model for HD [159,160]. The screen has yielded several highly potent compounds including C2–8, that was then shown to inhibit polyQ aggregation in cultured mammalian cells and intact neurons, and to rescue polyQ-mediated neurodegeneration in vivo [159]. The fact that several chemical compounds showed anti-aggregation properties in yeast led to successful pre-clinical studies in HD mouse models, demonstrating the value of yeast models for initial screening of toxicity modulators [160,161].

Intracellular antibodies (intrabodies) against Htt bind to huntingtin and prevent its misfolding and toxicity. Thus, intrabodies may be a useful gene-therapy approach to treatment of the disease. Disulfide bond-free single-domain intracellular antibody V_L_12.3 was engineered that inhibits aggregation and toxicity in the *S. cerevisiae* and neuronal cell culture models of HD [162]. These effects were later validated in some mouse models of HD [163,164] and strengthened the concept of using intrabodies as a therapeutic approach against HD.

By using a yeast deletion library, a set of gene deletions that suppress toxicity of a mutant Htt-103Q fragment has been discovered [157]. Unfortunately, this screening has not considered that some deletion strains from the collection have lost the Rnq1 prion, [*PIN^+^*], that is required for the Htt103Q cytotoxicity in the given yeast strain [148]. Indeed, it turned out that some deletion derivatives that have lost [*PIN^+^*] were false positives in the screen [165]. However, the most potent suppressor, deletion of a gene that encodes Bna4 (kynurenine 3-monooxygenase, KMO), an enzyme in the kynurenine pathway of tryptophan degradation, was not a result of [*PIN^+^*] loss. This enzyme has been linked directly to the pathophysiology of Huntington’s disease in humans [166]. In agreement, treatment with a small molecule inhibitor of KMO, Ro 61-8048, results in a partial amelioration of growth defects in Htt103Q-expressing yeast cells [157]. KMO inhibition leads to an altered product and intermediate profile of tryptophan degradation, reducing cellular stress and cell death [157,159]. The kynurenine pathway is now well-studied and discussed as a drug target for HD [166,167]. Further on, KMO inhibition has been extensively approached pharmacologically and chemically in pre-clinical rodent and Drosophila HD models [168,169].

A yeast HD model was also used to screen for the huntingtin aggregation/toxicity modifiers among the natural substances. For example, the polyphenol (−)-epigallocatechin-3-gallate (EGCG), a major bioactive component in green tea, has been identified as a potent suppressor and modulator of Htt aggregation and toxicity in yeast models [170]. This substance has become a promising candidate for healthy aging and promotes lifespan extension in worms, flies, and rodents [159,171,172,173,174].

In another high-throughput screen of natural products in a yeast HD model, actinomycin D was identified as a potent aggregation inhibitor [175]. It was demonstrated that applying a low dose of actinomycin D results in increased levels of certain Hsps (including Hsp104, Hsp70, and Hsp26) and enhanced binding of Hsp70 to the polyQ in yeast. The drug actinomycin D has many approved medical uses and could become an exciting drug lead in HD research.

Raspberry (*Rubus idaeus* var. Prestige) extracts were tested on different *S. cerevisiae* strains expressing disease proteins associated with Alzheimer’s, Parkinson’s, or Huntington’s disease [176]. Salidroside, a glycosylated phenol, displayed significant bioactivity against Huntington’s disease. Next, a metabolic route to salidroside was reconstructed in *S. cerevisiae* generating the yeast strain able to produce salidroside with the same positive effects as salidroside of natural origin [176]. The mechanism by which the *R. idaeus* polyphenol-enriched extract mediates cellular protection is associated with the removal of superoxide anions accumulated by the expression of HTT103Q-GFP.

## 9. Drug Discovery in Yeast Model of Parkinson’s Disease

Aggregation of alpha-synuclein (αSyn), a small 140-amino-acid protein, is a hallmark of Parkinson’s disease [177]. Yeast does not have an ortholog of αSyn, but several features of PD can be reproduced in yeast expressing human αSyn. In the first yeast model for PD, human αSyn was expressed in wild-type yeast cells. Expression of αSyn in yeast cells results in intracellular inclusions of αSyn, is toxic as reflected by growth inhibition, and can cause cell death [178]. Overexpression of αSyn inhibited cell growth in a αSyn dose-dependent manner [178]. Pathways that are associated with αSyn toxicity include vesicular trafficking, endocytosis, ubiquitin–proteasomal system, lipid metabolism, oxidative stress, mitochondria function, and autophagy [179].

Yeast was used for screens that resulted in the identification of several therapeutic candidates, rescuing αSyn aggregation and toxicity [180]. Two flavonoids, quercetin and epigallocatechin gallate, were identified as preventing αSyn toxicity in the presence of iron, reinforcing the role of oxidative stress in αSyn-initiated cellular degeneration [181]. Small molecules that rescue αSyn toxicity by stimulating function of the Rab GTPase, associated with PD, and/or increasing Rab1 levels were also obtained [182]. A screen of about 115,000 compounds in the yeast cells, expressing αSyn in a fusion with yellow fluorescent protein (YFP), identified a class of structurally related 1,2,3,4-tetrahydroquinolinones [183]. These compounds were found to reduce the formation of αSyn inclusions, re-establish ER-to-Golgi trafficking, and ameliorate the mitochondrial dysfunction [183]. It was also shown that the same small molecules are counteracting the toxicity of αSyn in nematodes and in primary rat neuronal midbrain cultures [183]. Cyclic peptides (CPs), natural product-like chemicals with potent bioactivity were also screened in a yeast PD model. Two related CPs—identified as reducing αSyn toxicity in yeast—also prevented dopaminergic neuron loss in the nematode, *Caenorhabditis elegans* [184]. In another screen, a N-aryl benzimidazole (NAB) was found to protect against αSyn toxicity not only in yeast but also in other models of PD (*C. elegans*, rat primary neuronal cultures and cortical neurons, differentiated from PD-patient-induced pluripotent stem cells [185]. These screens also revealed the conserved mode of action of this compound, which promotes endosomal transport via the E3 ubiquitin ligase, Rsp5/Nedd4, alleviating the dysfunctional endosomal and ER-to-Golgi vesicle trafficking promoted by αSyn [185]. Mannosylglycerate, a compatible solute typical of marine microorganisms thriving in hot environments, was found to reduce αSyn aggregation in a yeast model of PD [186]. Ascorbic acid, a natural antioxidant, was found to promote a significant reduction in the percentage of yeast cells bearing αSyn inclusions [187].

## 10. General and Specific Patterns of the Yeast Models for Anti-Amyloid Drug Discovery

The main feature of yeast models for neurodegenerative disorders, such as PD, HD and AD, is expression of a human disease hallmark protein, forming cross-β amyloid structures, in yeast cells. Aggregation (and in some cases, toxicity) of amyloidogenic proteins appears to show similar patterns in yeast and human cells. The advantage of yeast models includes unicellularity, rapid growth, easy cultivation techniques and a wide range of research tools available. Yeast is a eukaryotic organism with defined cellular compartments and similar systems of vesicular trafficking, a key component in neurological signaling linked to neurodegenerative disorders. The majority (although not all) of the key chaperone families modulating protein aggregation are conserved between yeast and humans. “Humanized” yeast models are extremely useful for the early steps in the discovery of candidate compounds that can be used for the development of a treatment against the disease. Screens for compounds preventing aggregation and toxicity of disease-specific proteins were performed and revealed potential leads which were then validated in animal models. Even despite the obvious fact that some of the physiological processes involved specifically in the neurobiology of Alzheimer’s, Huntington’s and Parkinson’s diseases cannot be recapped in this simple single-cell model, yeast assays have a unique property of efficiently targeting the mechanism of protein oligomerization/aggregation, a triggering factor in these diseases.

## 11. Conclusions

The development of effective therapies and preventive treatments for neurodegenerative diseases such as Alzheimer’s, Huntington’s and Parkinson’ diseases is still a great challenge, mainly because of insufficient knowledge of both molecular mechanisms of diseases, and environmental factors triggering and affecting these diseases. Yeast cells contain endogenous amyloid proteins (yeast prions), that cause easily detectable phenotypes and are efficiently employed for understanding the general mechanisms of amyloid formation and propagation (applying to both yeast and humans), identifying the pro- or anti-prion agents and conditions with a broad spectrum of action, and building the amyloid-specific detection tools. In this way, yeast models contribute to understanding of molecular foundation of the disease, identification of molecular targets and new compounds with therapeutic potentials.
